# Stabilization of Long-Looped i-Motif DNA by Polypyridyl Ruthenium Complexes

**DOI:** 10.3389/fchem.2019.00744

**Published:** 2019-11-05

**Authors:** Benjamin J. Pages, Sarah P. Gurung, Kane McQuaid, James P. Hall, Christine J. Cardin, John A. Brazier

**Affiliations:** ^1^School of Pharmacy, University of Reading, Reading, United Kingdom; ^2^Department of Chemistry, University of Reading, Reading, United Kingdom; ^3^Diamond Light Source, Didcot, United Kingdom

**Keywords:** i-motif, DNA, ruthenium, melting, stabilization, luminescence

## Abstract

A spectroscopic study of the interactions of Λ- and Δ-[Ru(phen)_2_(dppz)]^2+^ with i-motif DNA containing thymine loops of various lengths. In the presence of i-motifs, the luminescence of the Λ enantiomer was enhanced much more than the Δ. Despite this, the effect of each enantiomer on i-motif thermal stability was comparable. The sequences most affected by [Ru(phen)_2_(dppz)]^2+^ were those with long thymine loops; this suggests that long-looped i-motifs are attractive targets for potential transition metal complex drugs and should be explored further in drug design.

## Introduction

**Figure 1:**
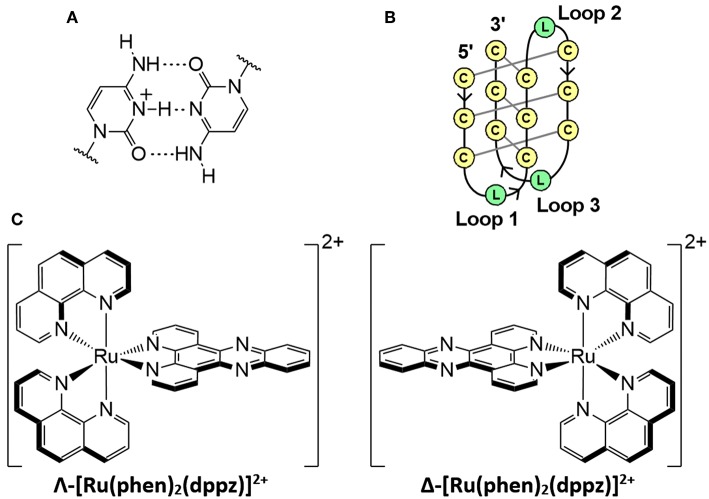
**(A)** Hemi-protonated cytosine^+^-cytosine base pair. **(B)** Schematic diagram of an intramolecular i-motif where C is cytosine and L represents the loop regions with any DNA base. **(C)** Enantiomers of the polypyridyl ruthenium complex; [Ru(phen)_2_(dppz)]^2+^.

The intercalated motif (i-motif) is a DNA structure containing intercalated cytosine^+^-cytosine base pairs between four strands (Figures 1A,B) (Gehring et al., 1993; Školáková et al., 2019). I-motifs were originally thought to only form in acidic pH due to the protonation of cytosine required; however, stable i-motif formation has been reported at alkaline (Zhou et al., 2010) and neutral pH (Day et al., 2013; Fujii and Sugimoto, 2015; Wright et al., 2017), as well as conditions mimicking physiological molecular crowding (Rajendran et al., 2010). Moreover, recent demonstrations of the presence of i-motifs in the nuclei of human cells (Dzatko et al., 2018; Zeraati et al., 2018), and their ability to inhibit DNA polymerase (Takahashi et al., 2017) has increased interest in their biological function (Abou assi et al., 2018). Sequences that are complementary to those that form G-quadruplexes have been shown to form i-motif structures in the promoter regions of several cancer genes (Brooks et al., 2010; Brazier et al., 2012; Li et al., 2016). These include the transcription factors that code the cellular myelocytomatosis (c-Myc) (Mathur et al., 2004) and B-cell lymphoma-2 (Bcl-2) oncogenes, the latter of which is overexpressed in some cancers and may be underexpressed in some neurodegenerative diseases (Bar-Am et al., 2005; Knight et al., 2019). The transcription factor hnRNP LL reportedly binds to i-motifs, which suggests that the i-motif acts as a recognition site for the activation of transcription of Bcl-2(Kang et al., 2014).

The unique structure and potential biological roles of the i-motif makes it an attractive binding target for small molecules, particularly if the binding results in stabilization. For example, the porphyrin TMPyP4 reportedly binds to i-motifs similar in structure to the human telomeric sequence [5′-(C_3_TAA)_3_C_3_-3′] (Fedoroff et al., 2000). A variety of other i-motif-binding ligands have been reported, including carbon nanotubes (Li et al., 2006), bis-acridines (Alberti et al., 2001), mitoxantrone (Wright et al., 2016), crystal violet (Ma et al., 2011), and derivatives of thiazole orange (Sheng et al., 2017) and coumarin (Satpathi et al., 2019). An under-researched family of ligands in the field of i-motifs is transition metal complexes, of which i-motif binding studies are limited (Shi et al., 2010a,b; Lu et al., 2015). Metal complexes have been studied as DNA binders for decades due to their uses as therapeutic and diagnostic agents (Metcalfe and Thomas, 2003; Pages et al., 2015; Deo et al., 2016). In particular, polypyridyl ruthenium complexes have been extensively researched for medicinal purposes due to their high stability, affinity for DNA, and luminescence properties (Gill and Thomas, 2012; Deo et al., 2016; Poynton et al., 2017). The complex [Ru(phen)_2_(dppz)]^2+^ (Figure 1C, where phen = 1,10-phenanthroline; dppz = dipyrido[3,2-*a*:2′3′-*c*]phenazine) and derivatives demonstrate a DNA “light switch” effect, in which luminescence is greatly enhanced when bound to DNA (Friedman et al., 1990; Cardin et al., 2017). The equilibrium between dark and emissive states is influenced by both changes in solvent environment around the ancillary phen ligands and changes in the hydrogen bonding of solvent molecules with the pyrazine nitrogen atoms of the dppz ligand (Chantzis et al., 2013; Véry et al., 2014). The emissive state occurs during intercalative binding of [Ru(phen)_2_(dppz)]^2+^ with DNA (Hartshorn and Barton, 1992; Olofsson et al., 2004). Solution and crystallographic studies have revealed that this complex and its derivatives bind to duplex and G-quadruplex DNA (Wilson et al., 2013; Hall et al., 2016; McQuaid et al., 2019a), and can inhibit telomerase activity (Yu et al., 2012). Preliminary work has demonstrated non-specific binding between i-motifs and *rac-*[Ru(phen)_2_(dppz)]^2+^ (*rac*-Ru) (Shi et al., 2010a,b).

We have previously demonstrated that the stability of i-motifs is influenced by loop length, with longer loops resulting in lower overall stability and vice-versa; this is likely due to the differences in flexibility of the loop regions (Gurung et al., 2015). We speculate that these regions are more desirable binding sites as they aren't as tightly packed as the intercalated cytosine core. In fact, the nature of the lateral loops of the Bcl-2 i-motif was determined to be vital to the binding potency of IMC-48 (Kang et al., 2014). Here we report the first study of interactions between enantiomerically resolved ruthenium complexes and i-motifs with various loop lengths. We have used synchrotron radiation circular dichroism (SRCD), UV, and luminescence spectroscopy to probe the binding of Λ-[Ru(phen)_2_(dppz)]^2+^ (Λ-Ru) and Δ-[Ru(phen)_2_(dppz)]^2+^ (Δ-Ru) to a series of i-motif sequences. These consisted of a block of three paired cytosines and combinations of long and short thymine loops. Loop lengths were either uniform (C_3_T_X_, e.g., C_3_T_4_) or combinations of 3 and 8 thymines (C_3_T_XXX_ e.g., C_3_T_383_), with C_3_T_3_/C_3_T_333_, and C_3_T_8_/C_3_T_888_ being in both “groups” (Table 1).

**Table 1:** DNA melting temperatures (*T*_M_) of the C_3_T_X_ and C_3_T_XXX_ sequences (1 μM ss) with and without 1 equiv. Λ-Ru or Δ-Ru.

**Label**	**Sequence 5^′^ → 3^′^**	**Native**	**Λ-Ru**		**Δ-Ru**	
		***T*_**M**_**	***T*_**M**_**	**Δ*T*_**M**_**	***T*_**M**_**	**Δ*T*_**M**_**
C_3_T_3/333_	(CCCTTT)_3_CCC	60.0	60.3	+0.3	60.3	+0.3
C_3_T_4_	(CCCTTTT)_3_CCC	59.6	58.9	−0.7	58.7	−0.9
C_3_T_5_	(CCCTTTTT)_3_CCC	51.7	51.4	−0.3	51.4	−0.3
C_3_T_6_	(CCCTTTTTT)_3_CCC	47.0	48.4	+1.4	48.2	+1.2
C_3_T_7_	(CCCTTTTTTT)_3_CCC	41.9	45.8	+3.9	45.7	+3.8
C_3_T_8/888_	(CCCTTTTTTTT)_3_CCC	37.8	44.1	+6.3	43.2	+5.4
C_3_T_338_	CCCTTTCCCTTTCCCTTTTTTTTCCC	49.1	50.8	+1.7	50.5	+1.4
C_3_T_383_	CCCTTTCCCTTTTTTTTCCCTTTCCC	55.3	55.2	−0.1	55.0	−0.3
C_3_T_388_	CCCTTTCCCTTTTTTTTCCCTTTTTTTTCCC	46.1	48.1	+2.0	47.2	+1.1
C_3_T_833_	CCCTTTTTTTTCCCTTTCCCTTTCCC	49.5	51.0	+1.5	50.8	+1.3
C_3_T_838_	CCCTTTTTTTTCCCTTTCCCTTTTTTTTCCC	39.8	45.7	+6.0	45.6	+5.9
C_3_T_883_	CCCTTTTTTTTCCCTTTTTTTTCCCTTTCCC	46.6	48.8	+2.2	47.8	+1.2

*Values are in degrees Celsius with a standard deviation of ±~0.1–0.6°C. Standard deviation for individual measurements are shown in Table S3.1*.

## Materials and Methods

### Materials

Λ- and Δ-[Ru(phen)_2_dppz]^2+^ were synthesized and resolved through our previously reported methods; full details are included in the Supporting Information (Ortmans et al., 2004; McQuaid et al., 2019a). Unless otherwise stated, all materials and chemicals were sourced from Sigma-Aldrich (Merck) or Honeywell research chemicals. Oligonucleotides were obtained from Eurogentec (RP-HPLC purified) and used without further purification. All solvents were obtained at HPLC grade and used without further purification.

### Solution Preparation and Annealing

Initial stock solutions of the ruthenium complexes and oligonucleotides were made in water and checked for concentration using the extinction coefficient of 20 000 M^−1^ cm^−1^ at 440 nm for the former, and for the latter, the Eurogentec-provided extinction coefficients at 260 nm, calculated using the nearest-neighbor model. The stocks were then diluted with buffer and combined to form solutions with either a 1:0 or 1:1 ratio of DNA strand to ruthenium complex. Annealing of the oligonucleotides, both with and without ruthenium complex present, was achieved by incubating the buffered solution at 90°C for 5 min, and then allowing it to cool to room temperature overnight. Preliminary experiments showed that adding ruthenium complex before or after i-motif annealing did not affect the melting temperature (Figure S3.1), and so all ruthenium additions in this study were done pre-annealing.

### Synchrotron Radiation Circular Dichroism

Samples consisted of the oligonucleotide [100 μM single stranded (ss)] and ruthenium complex (100 μM) in 20 mM sodium cacodylate buffer at pH 5. The concentration of buffer was lowered relative to other experiments to obtain the lowest data resolution cut-off. CD spectra were recorded at 20 °C between 195 and 350 nm with a 1 nm increment. Experiments were performed in a 0.01 cm pathlength cuvette, on beamline B23 at Diamond Light Source Ltd.

### UV Melting

UV melting experiments were carried out using Agilent Cary 100 with a temperature controlled six-cell changer. Samples consisted of the oligonucleotide (1 μM ss) and either 0 or 1 molar equivalent of the ruthenium complex. The buffer consisted of 50 mM sodium cacodylate at a pH of either 5 or 8. Absorption was recorded at 260 and 295 nm at 1 °C intervals between 20–90°C, with a temperature change rate of 0.5 °C/min in a 1 cm pathlength quartz cuvette. Melting curves were generated from this data. To determine the melting temperature, the curves were fitted with a sigmoidal function: $y=\frac{H}{1+\exp\left[-St\times \left(T-T_{M}\right)\right]}+S\;$where the constants are *H, St, S* and *T*_M_: the height, steepness, starting point and inflexion point (melting temperature) of the function, respectively, and the variable is *T*, the temperature. This function was generated for each curve through minimization of the sum of residuals between the raw data and model. The fit was applied to the region that best represented a sigmoid. Experiments were performed in triplicate and the results averaged.

### Luminescence Spectroscopy

Luminescence spectroscopy measurements were performed using a 1 cm pathlength quartz cell at room temperature. Samples consisted of oligonucleotides and ruthenium complex with a final concentration of 20 μM each (ss DNA), dissolved in 50 mM sodium cacodylate buffer (pH 5 or 8). Emission spectra were measured between 550 and 875 nm with an excitation wavelength of 440 nm.

## Results and Discussion

### Synchrotron Radiation Circular Dichroism

SRCD spectra of the C_3_T_X_ sequences were obtained with and without the presence of *rac*-Ru at pH 5 (Figure S2.1). *Rac*-Ru had to be used here as the CD signal of Λ-Ru and Δ-Ru would obscure that of the i-motif. Only minor changes in CD spectra were observed with addition of *rac-*Ru, demonstrating that the i-motif structure still forms in the presence of ruthenium complexes. This is supported by the UV melting profiles of both native and ruthenium-bound C_3_T_X_ and C_3_T_XXX_, which show characteristic hyperchromicity at 260 nm and hypochromicity at 295 nm (Figures S3.2–5) (Phan and Mergny, 2002).

### UV Melting

**Figure 2:**
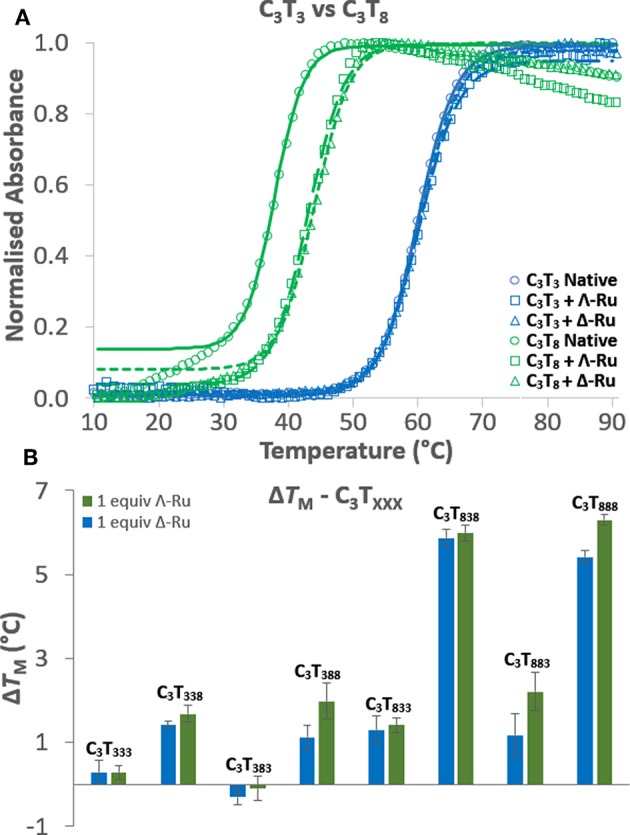
**(A)** UV melting curves of C_3_T_3_ (blue) and C_3_T_8_ (green) in their native forms (circles) and in the presence of Λ-Ru (squares) or Δ-Ru (triangles). **(B)** Comparison of the Δ*T*_M_ induced by Λ-Ru (green) and Δ-Ru (blue) for C_3_T_XXX_.

UV melting experiments revealed that for C_3_T_X_ sequences with loops of 6 thymines or less, there was no appreciable difference (<1.5°C) in melting temperature (*T*_M_) with one equivalent of Λ-Ru or Δ-Ru, while stabilization of ~4°C occurred for C_3_T_7_ and ~6°C for C_3_T_8_ (Table 1, Figure 2A, and Figure S3.7). This result suggests that the longer loops have cavities that allow binding of ruthenium complexes, resulting in stabilization. For C_3_T_XXX_, sequences with longer loops 1 and 3 experienced the most stabilization with ruthenium, while a long loop 2 did not affect stabilization (Figure 2B, Table 1). For example, the Δ*T*_M_ of C_3_T_838_ was ~6°C, while C_3_T_383_ was not stabilized. Interestingly, the sequences with the lowest native stability were those that were stabilized by Λ-Ru and Δ-Ru the most. There was little difference in the effect of Λ-Ru and Δ-Ru on sequence stability, although Λ-Ru did occasionally produce higher Δ*T*_M_ values. These experiments were repeated at pH 8 for C_3_T_3_ and C_3_T_8_; a melting curve was not observed, implying an absence of i-motif structure (Figure S3.6).

### Luminescence

**Figure 3:**
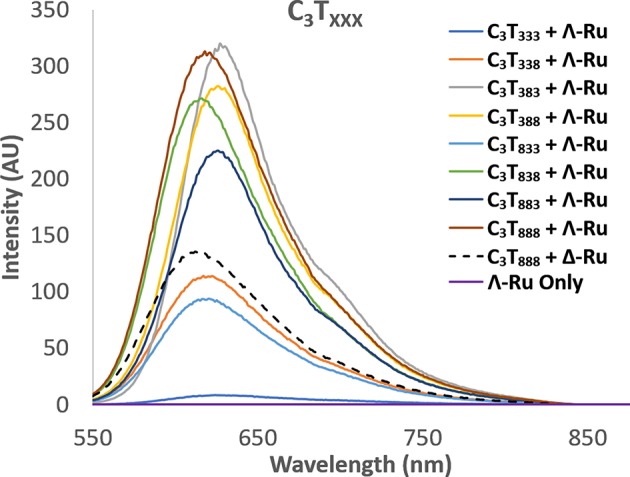
Luminescence spectra of Λ-Ru (20 μM) with C_3_T_XXX_ (20 μM ss). The spectrum of Δ-Ru with C_3_T_888_ is also shown for comparison (broken line).

Enhancement of luminescence was observed for Λ-Ru and Δ-Ru when bound to all i-motif sequences, aside from C_3_T_3_, for which little enhancement occurred (Figure 3, Figure S4.1). For non-C_3_T_3_ sequences, emission was much higher for Λ-Ru than for Δ-Ru, meaning that the solvent environment and pyrazine nitrogen exposure were more favorable for luminescence of Λ-Ru. Without the presence of DNA, the emission of each complex was very low (Figure 3, Figure S4.1). For C_3_T_X_, emission generally increased with loop length, with a large increase for C_3_T_7_ and C_3_T_8_ (Figure S4.1). For C_3_T_XXX_, trends differed between Λ-Ru and Δ-Ru. For Δ-Ru, C_3_T_838_ and C_3_T_888_ produced the highest emission (>300x Ru alone), C_3_T_333_ showed relatively little enhancement (20x Ru alone), and the other sequences showed relatively moderate enhancement (100-140x Ru alone). For Λ-Ru, emission was more varied per sequence and was correlated with loop length (Figure 3). A long loop 2 resulted in more than double the emission of a long loop 1 or 3, as evidenced by the higher emission of C_3_T_383−_ (600x Ru alone) relative to C_3_T_338_, C_3_T_833_ (200-250x Ru alone), and C_3_T_838_ (550x Ru alone).

In addition to luminescence enhancement, the emission maxima shifted depending on the sequence. For Δ-Ru, λ_max_ was generally blue-shifted with increasing loop length (Table S4.1, Figure S4.3). This correlated positively with emission intensity, which has been observed for ruthenium complexes that undergo stacking with G-quadruplex DNA (Wilson et al., 2010). However, for Λ-Ru, there are few trends relating λ_max_ to loop length, or to intensity (Figure S4.3); this overall suggests while Δ-Ru is likely to undergo base stacking with i-motif loops, Λ-Ru may also interact through different modes. The luminescence of Λ-Ru and Δ-Ru with C_3_T_3_ and C_3_T_8_ was also recorded at pH 8; interestingly, emission enhancement still occurred despite the lack of i-motif structure. Relative to their i-motif forms, C_3_T_3_ and C_3_T_8_ produced higher and lower enhancement, respectively (Figure S4.2). Luminescence enhancement of [Ru(phen)_2_(dppz)]^2+^ due to interactions with single stranded sequences without tertiary structure has been previously reported (Coates et al., 2001), and could explain this phenomenon.

### Melting vs. Luminescence

**Figure 4:**
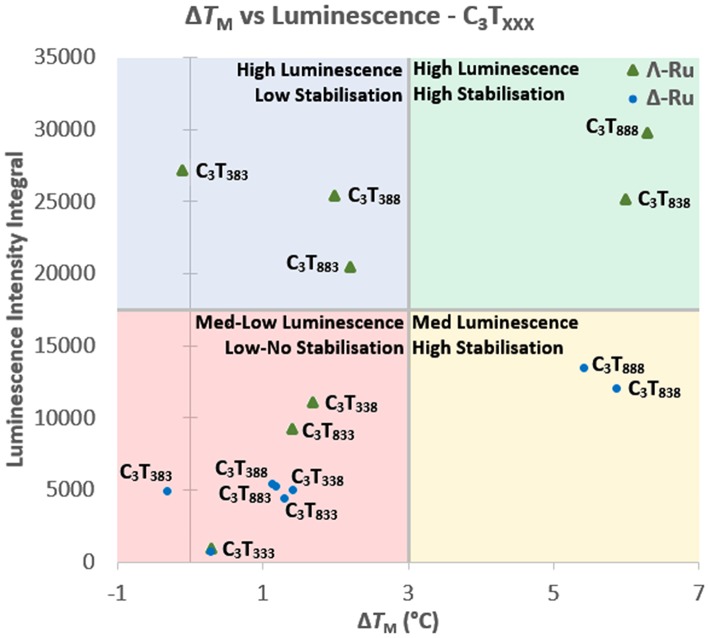
Comparison of the integrated luminescence intensity and Δ*T*_M_ of Λ-Ru (green triangles) and Δ-Ru (blue circles) when bound with C_3_T_XXX_. The graph is quartered into sections based upon the relative luminescence enhancement and thermal stabilization for each i-motif-ruthenium combination.

When comparing the UV melting and luminescence data for these complexes, some trends do emerge. For C_3_T_X_, the binding of Λ-Ru and Δ-Ru to longer-looped sequences resulted in both higher Δ*T*_M_ and higher ruthenium emission (Figure S5.1). For shorter loops, luminescence generally increased with increasing length, but melting stabilization did not. For the C_3_T_XXX_ series, there are some trends relating emission, *T*_M_ and the position of the long loops (Figure 4). The luminescence of Λ-Ru indicates that it binds to longer loops irrespective of where they are positioned, but the *T*_M_ data only shows stabilization when bound to loops 1 or 3. Λ-Ru demonstrated high luminescence with C_3_T_383_, but no stabilization, whereas interaction with C_3_T_838_ and C_3_T_888_ resulted in high luminescence and stabilization. C_3_T_333_ was not stabilized and did not enhance luminescence. The other sequences were slightly stabilized and produced medium-low luminescence enhancement (Figure 4). Overall, the presence of Λ-Ru with longer loops 1 and 3 resulted in increased stability and luminescence, while only luminescence was increased with a long loop 2. Δ-Ru did not follow the same C_3_T_XXX_ trends as Λ-Ru, aside from low luminescence and Δ*T*_M_ for C_3_T_333_ (Figure 4). Luminescence and Δ*T*_M_ were only notable for C_3_T_888_ and C_3_T_838_, while these values were medium-low for the remaining sequences. Unlike Λ-Ru, the interactions of Δ-Ru with a long loop 2 did not increase luminescence relative to loops 1 or 3, despite resulting in approximately the same thermal stabilization as Λ-Ru.

## Conclusion

In this study we have demonstrated that the length and position of i-motif thymine loops not only impacts the native structure, but also the degree of stabilization by Λ-Ru and Δ-Ru. These complexes do not stabilize short-looped sequences, but do stabilize the relatively less stable, long-looped i-motifs. It is possible that the longer loops are more flexible, and that they may form T-T hairpins; we recently reported binding of Λ-[Ru(tetraazaphenanthrene)_2_(dppz)]^2+^ to mismatched T-T base pairs and speculate something similar may be occurring here in the longer i-motif loops (McQuaid et al., 2019b). The luminescence blue-shifting implies that base stacking and perhaps other modes may contribute to ruthenium-loop interactions, but further spectroscopic and crystallographic experiments are required to elucidate the true binding behavior. Overall, these results demonstrate that i-motif sequences with longer loops are potential transition metal drug targets for therapeutic and diagnostic purposes.

## Data Availability Statement

The raw data supporting the conclusions of this manuscript will be made available by the authors, without undue reservation, to any qualified researcher.

## Author Contributions

BP led the writing of the manuscript and performed UV and luminescence experiments. SG performed the SRCD experiments, supported by JH. BP and SG each were responsible for analysis of their collected data. KM synthesized and resolved the ruthenium enantiomers. CC and JB conceived the project and supervised the work. All authors contributed to the writing of the manuscript.

### Conflict of Interest

The authors declare that the research was conducted in the absence of any commercial or financial relationships that could be construed as a potential conflict of interest.
