# Supplementary material for: Stabilization of Long-Looped i-Motif DNA by Polypyridyl Ruthenium Complexes
Source: Front Chem. 2019 Nov 5;7:744. doi: 10.3389/fchem.2019.00744 (PMC6848161; doi:10.3389/fchem.2019.00744)
Supplement: Supplementary file 1 [file Data_Sheet_1.PDF]

Supporting Information

1. Synthesis and chiral resolution of Λ- and Δ-$[Ru(phen)_2dppz]^{2+}$

The synthesis and chiral resolution of $[Ru(phen)_2dppz]^{2+}$ was achieved through a method similar to that recently described.(McQuaid et al., 2019) $Ru(phen)_2Cl_2$ (80 mg, 0.15 mmol) and dppz (30 mg, 15 mmol) were suspended in an aqueous ethanol solution (7 mL, 1:1) within a CM microwave tube (10 mL capacity). The solution was degassed with Ar for 10 minutes before sealing and installation into the synthetic microwave. The reaction solution was irradiated for 40 minutes at 150 °C using 140 W. The resultant red/brown solution was cooled and filtered under vacuum. The target compound was precipitated from the filtrate through dropwise addition of an aqueous solution of saturated KPF_6, filtered under vacuum, washed with cold water (2 x 2 mL), and air-dried. The crude product was then dissolved in the minimum amount of acetonitrile (~5 mL), water (10 mL) was added, and the mixture transferred to a beaker containing Amberlite ion exchange resin (IRA-400, Cl^- form, 2.4 g). The mixture was covered and gently stirred for 16 hours to convert the product to the chloride salt. The resin was then removed through gravity filtration and the solvent removed through rotary evaporation. Purification was achieved through flash chromatography on an aqueous Sephadex C-25 column using 0.2 M aqueous NaCl as the mobile phase. The orange/red fractions were combined and KPF_6 was again added to precipitate the crude product. Conversion to the chloride salt was then done through the same method to yield the racemic complex as a red/brown microcrystalline solid (98 mg, 0.12 mmol, 80%). Preparative scale chiral resolution of the product was achieved using a Hitachi Primeaide HPLC arrangement equipped with a CF6 LARIHC cyclofructan based chiral column (internal dimensions; 10 x 250 mm) supplied by AZYP separations; LLC (Arlington, Texas, US). The mobile phase was a solution comprising of 60:40:4:1.6 methanol:acetonitrile:triethylamine:acetic acid. The racemic complex was dissolved in the mobile phase at a concentration of approx. 25 mg/mL, and each injection was 200 uL. Separation was performed at a flow rate of 5 mL/min. Due to the small volume of the column, five separations were performed to obtain a reasonable amount of resolved product. The first (Δ-enantiomer) and second (Λ-enantiomer) eluents were collected in 15 mL falcon tubes. These solutions were reduced under pressure using a DNA concentrator at 40 °C for 16 h. 2-3 mL of saturated KPF_6 was then added to each tube and the solutions reduced under pressure again to fully remove organic solvent. At this point the products had precipitated as hexafluorophosphate salts, which were isolated through vacuum filtration and washed with water (5 x 5 mL). The products were then converted to their chloride salt as previously described to produce the final product as a red/brown solid. Optical purity was confirmed through analytical chiral HPLC and circular dichroism.

2. Synchrotron Radiation Circular Dichroism Spectra

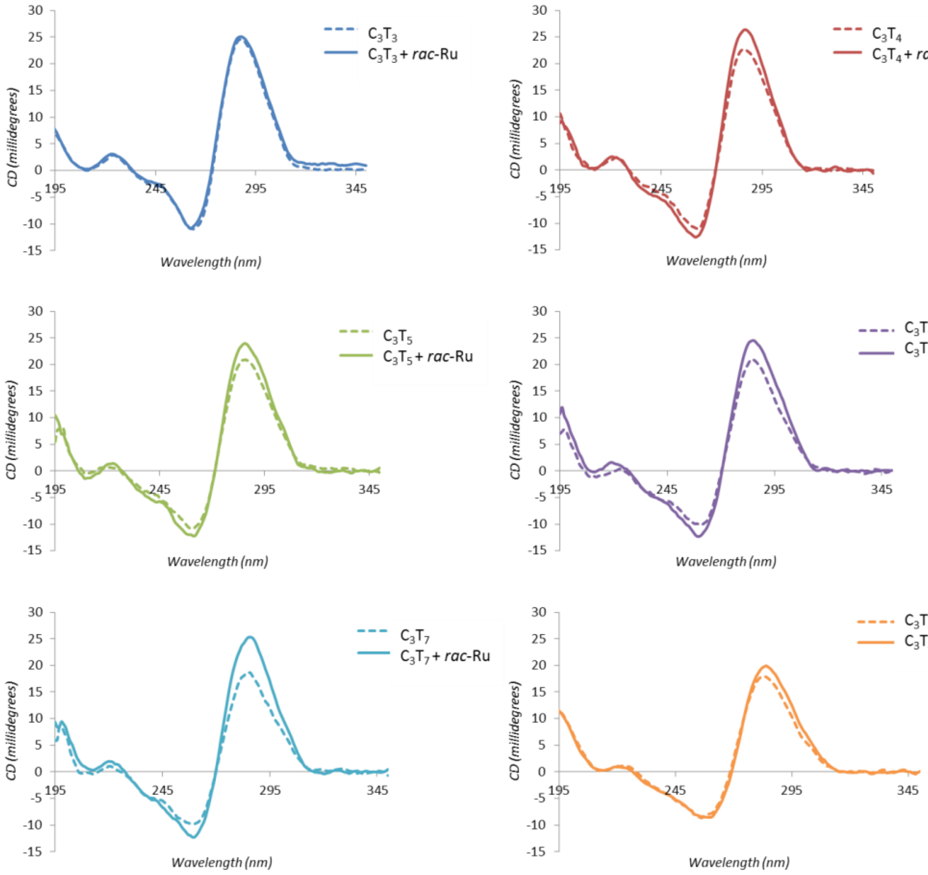

Figure S2.1. Synchrotron radiation CD spectra of C_3T_X (100 µM ss) with and without *rac*-[Ru(phen)$_2$dppz]$^{2+}$ (100 µM). Buffer consisted of 20 mM sodium cacodylate at pH 5.

3. UV Melting Data

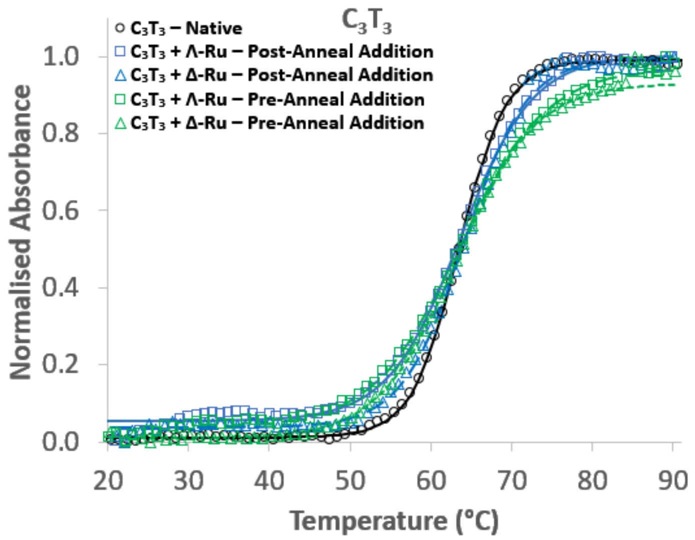

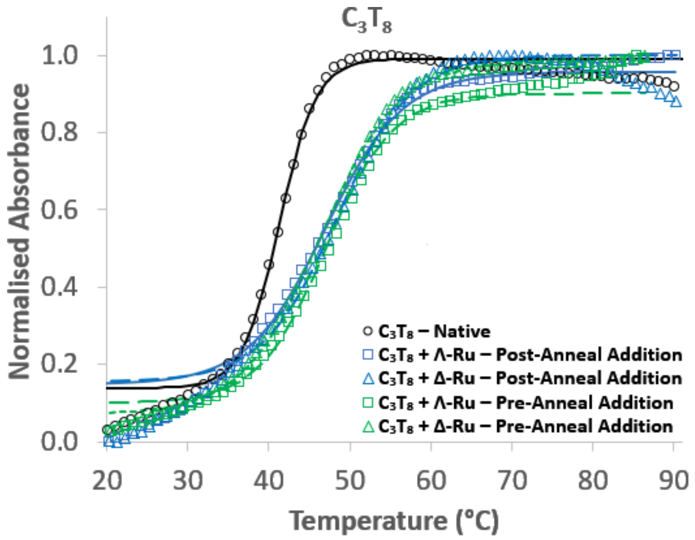

Figure S3.1. Comparison of normalised UV melting curves of C_3T_3 (top) and C_3T_8 (bottom) (1 µM ss) at 260 nm, showing native DNA (black circles), DNA with one equiv. Λ-Ru (squares) or Δ-Ru (triangles) added after DNA annealing (blue) or before DNA annealing (green). Fitted sigmoidal curves are also displayed for each sample in the corresponding colour. Buffer consisted of 50 mM sodium cacodylate at pH 5.

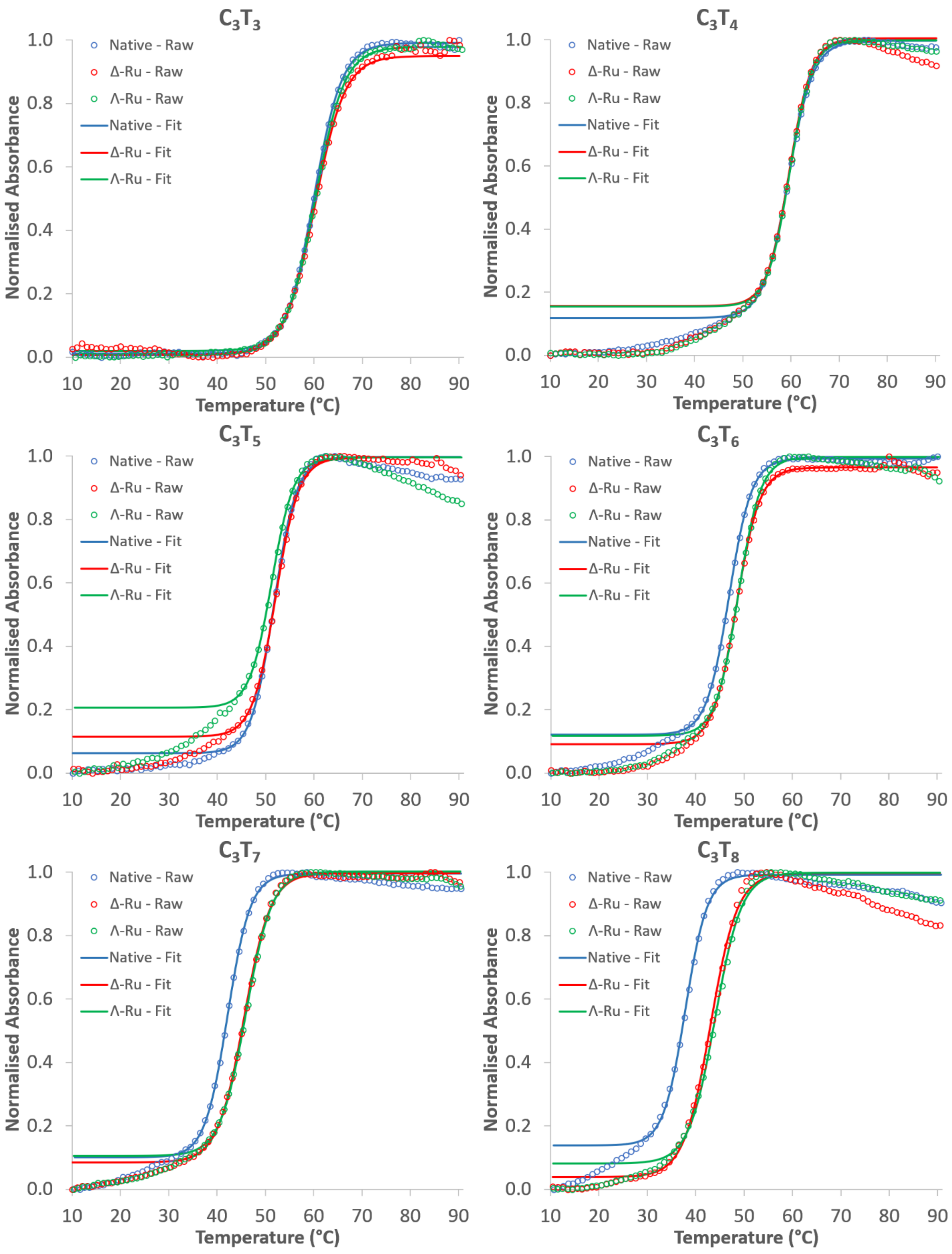

Figure S3.2. Comparison of normalised UV melting curves at 260 nm, showing raw data (hollow circles) and fitted sigmoidal model used to calculate the melting temperature (solid lines). Samples consisted of C_3T_X (1 µM), either native (blue), with Λ-Ru (1 µM, green) or with Δ-Ru (1 µM, red). Buffer consisted of 50 mM sodium cacodylate at pH 5.

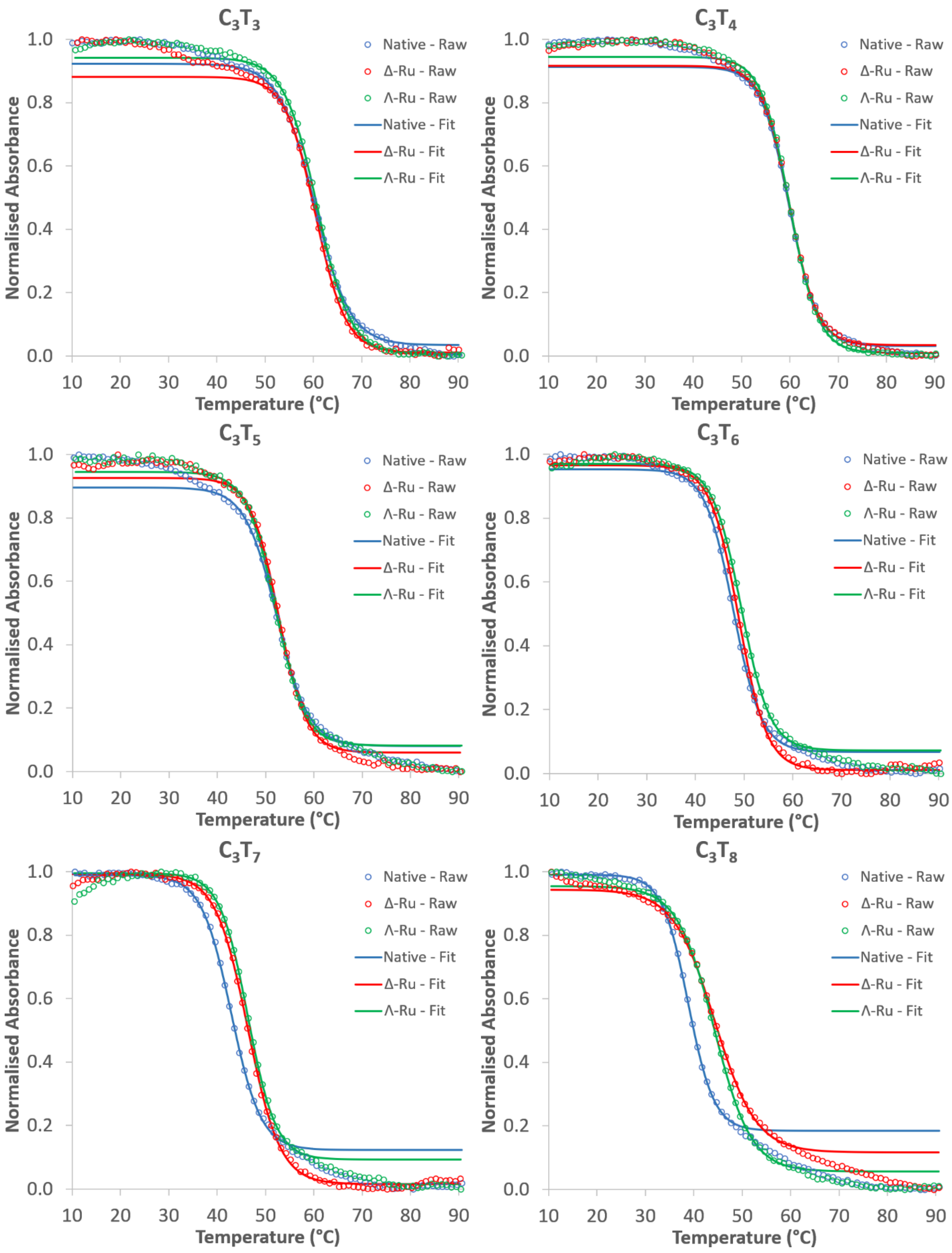

Figure S3.3. Comparison of normalised UV melting curves at 295 nm, showing raw data (hollow circles) and fitted sigmoidal model used to calculate the melting temperature (solid lines). Samples consisted of C_3T_X (1 µM), either native (blue), with Λ-Ru (1 µM, green) or with Δ-Ru (1 µM, red). Buffer consisted of 50 mM sodium cacodylate at pH 5.

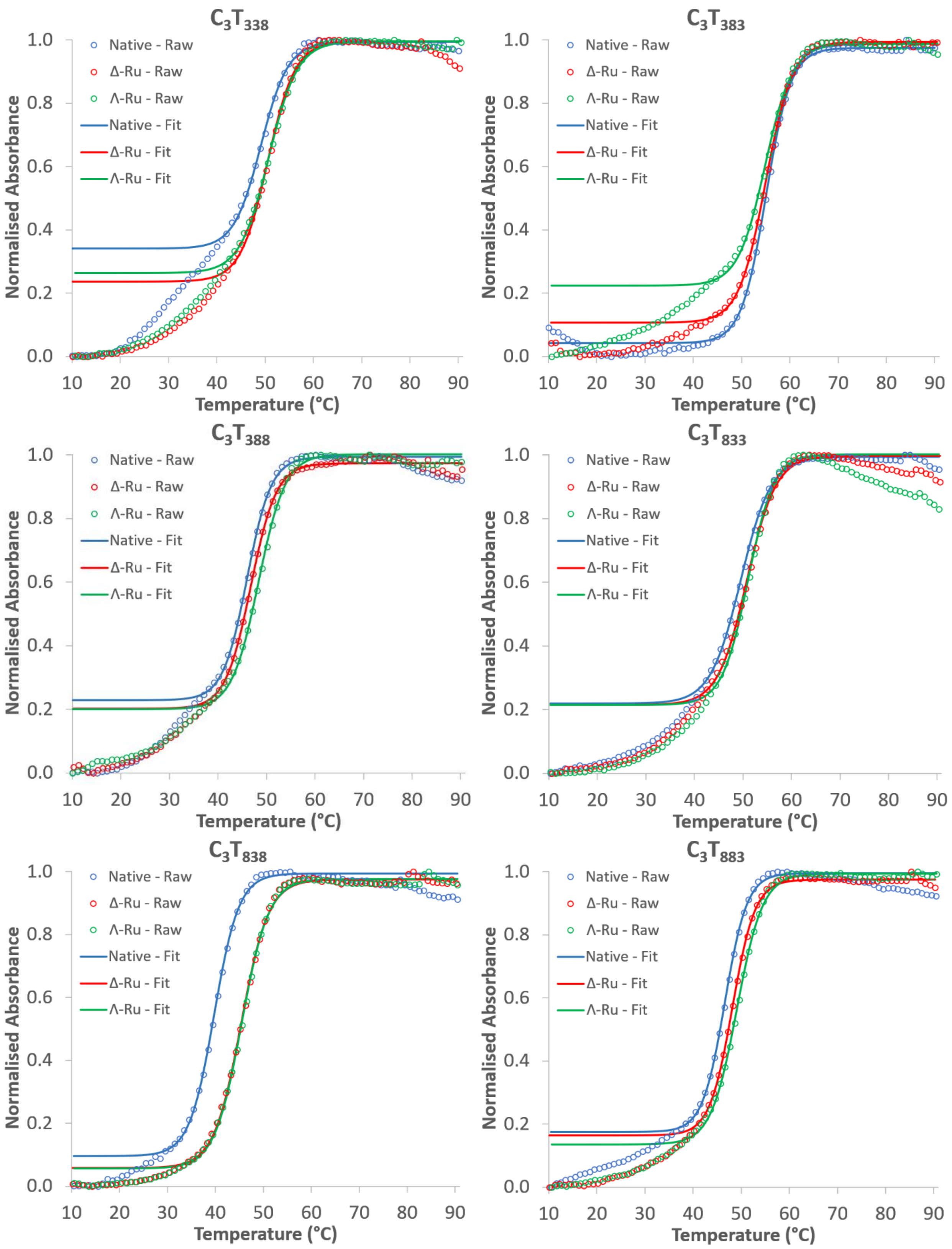

Figure S3.4. Comparison of normalised UV melting curves at 260 nm, showing the raw data (hollow circles) and fitted sigmoidal model used to calculate the melting temperatures (solid lines). Samples consisted of C_3T_{XXX} (1 µM), either native (blue), with Λ-Ru (1 µM, green) or with Δ-Ru (1 µM, red). Buffer consisted of 50 mM sodium cacodylate at pH 5.

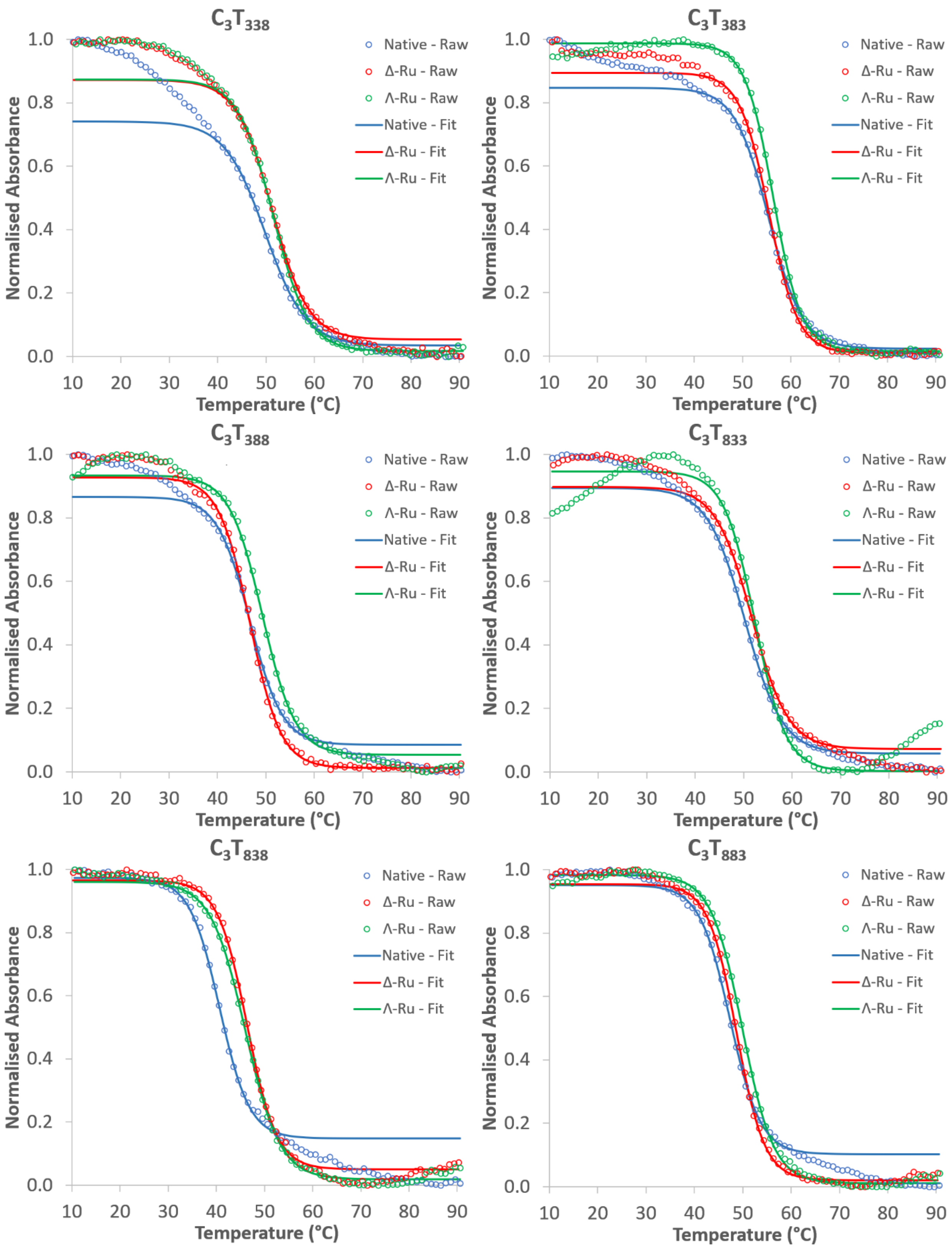

Figure S3.5. Comparison of normalised UV melting curves at 295 nm, showing the raw data (hollow circles) and fitted sigmoidal model used to calculate the melting temperatures (solid lines). Samples consisted of C_3T_{XXX} (1 µM), either native (blue), with Λ-Ru (1 µM, green) or with Δ-Ru (1 µM, red). Buffer consisted of 50 mM sodium cacodylate at pH 5.

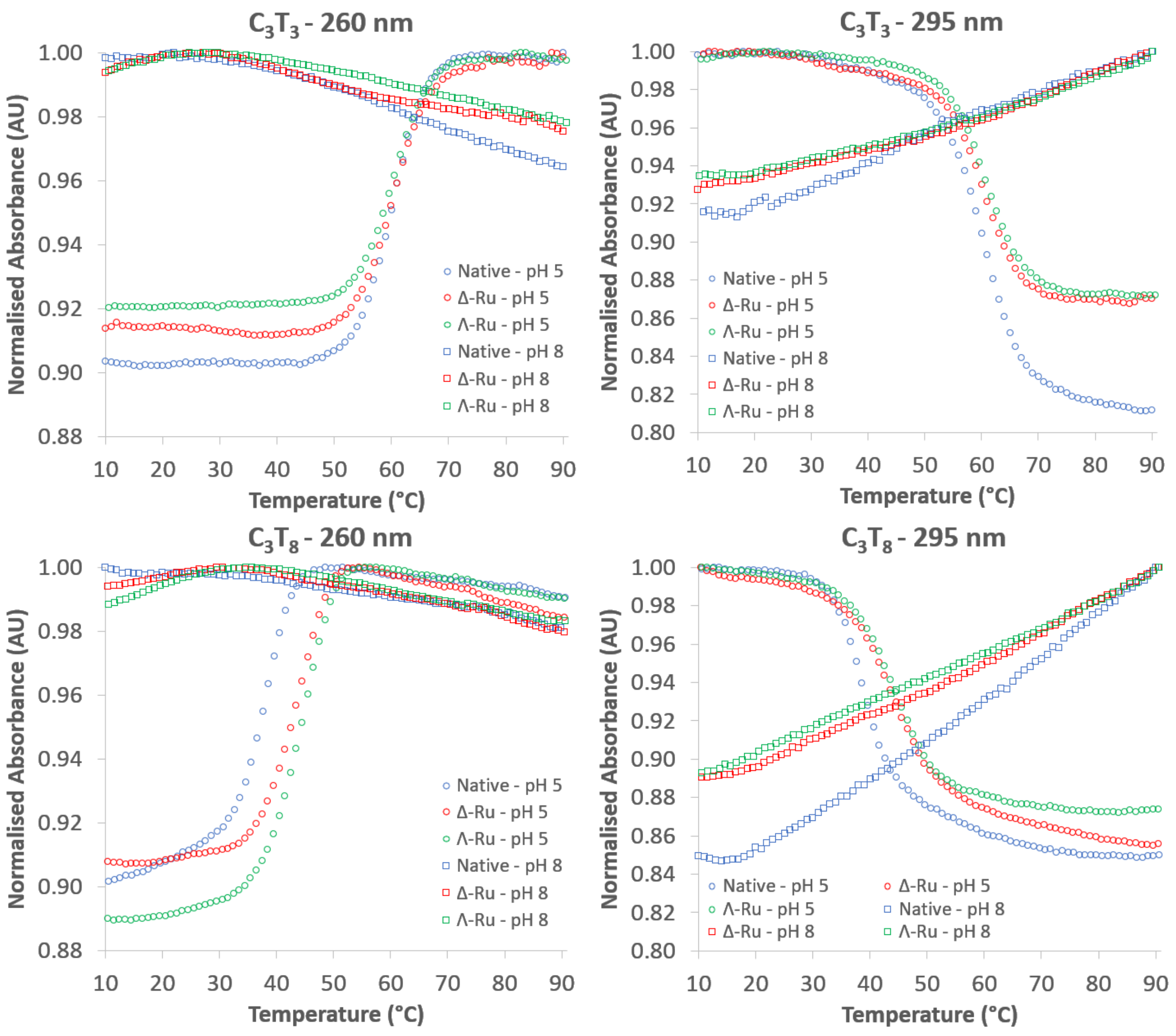

Figure S3.6. Comparison of normalised UV melting curves of C_3T_3 (top) and C_3T_8 (bottom) at 260 nm (left) and 295 nm (right) at pH 5 (circles) and pH 8 (squares). At pH 8 there are no melting curves present and the opposite chromicity to the pH 5 samples is observed, confirming the lack of i-motif structure. Samples consisted of C_3T_3 or C_3T_8 (1 µM), either native (blue), with Λ-Ru (1 µM, green) or with Δ-Ru (1 µM, red). Buffer consisted of 50 mM sodium cacodylate at pH 5 or 8.

Table S3.1: Full table of T_M values as per Table 1, with standard deviation included (1 sig. fig.).

Label	Sequence 5'→3'	Native	Λ-Ru		Δ-Ru	
		T_m	T_m	ΔT_m	T_m	ΔT_m
C_3T_3	$(CCCTTT)_3CCC$	60.0 ± 0.2	60.25 ± 0.02	+0.3 ± 0.3	60.3 ± 0.3	+0.3 ± 0.3
C_3T_4	$(CCCTTTT)_3CCC$	59.6 ± 0.1	58.9 ± 0.5	-0.7 ± 0.5	58.7 ± 0.6	-0.9 ± 0.6
C_3T_5	$(CCCTTTTT)_3CCC$	51.71 ± 0.02	51.4 ± 0.5	-0.3 ± 0.5	51.4 ± 0.5	-0.3 ± 0.5
C_3T_6	$(CCCTTTTTT)_3CCC$	47.0 ± 0.1	48.4 ± 0.5	+1.4 ± 0.5	48.2 ± 0.4	+1.2 ± 0.4
C_3T_7	$(CCCTTTTTTT)_3CCC$	41.9 ± 0.2	45.8 ± 0.1	+3.9 ± 0.2	45.7 ± 0.1	+3.8 ± 0.2
C_3T_8	$(CCCTTTTTTTT)_3CCC$	37.8 ± 0.1	44.1 ± 0.1	+6.3 ± 0.1	43.2 ± 0.1	+5.4 ± 0.1
C_3T_{338}	CCCTTTCCCTTTCCCTTTTTTTTCCC	49.08 ± 0.04	50.8 ± 0.2	+1.7 ± 0.2	50.50 ± 0.07	+1.40 ± 0.08
C_3T_{383}	CCCTTTCCCTTTTTTTTCCCTTTCCC	55.3 ± 0.2	55.2 ± 0.2	-0.1 ± 0.3	55.0 ± 0.1	-0.3 ± 0.2
C_3T_{388}	CCCTTTCCCTTTTTTTTCCCTTTTTTTTCCC	46.08 ± 0.07	48.1 ± 0.4	+2.0 ± 0.4	47.2 ± 0.3	+1.1 ± 0.3
C_3T_{833}	CCCTTTTTTTTCCCTTTCCCTTTCCC	49.5 ± 0.1	51.0 ± 0.1	+1.5 ± 0.2	50.8 ± 0.3	+1.3 ± 0.3
C_3T_{838}	CCCTTTTTTTTCCCTTTCCCTTTTTTTTCCC	39.75 ± 0.03	45.7 ± 0.2	+6.0 ± 0.2	45.6 ± 0.2	+5.9 ± 0.2
C_3T_{883}	CCCTTTTTTTTCCCTTTTTTTTCCCTTTCCC	46.63 ± 0.04	48.8 ± 0.5	+2.2 ± 0.5	47.8 ± 0.5	+1.2 ± 0.5

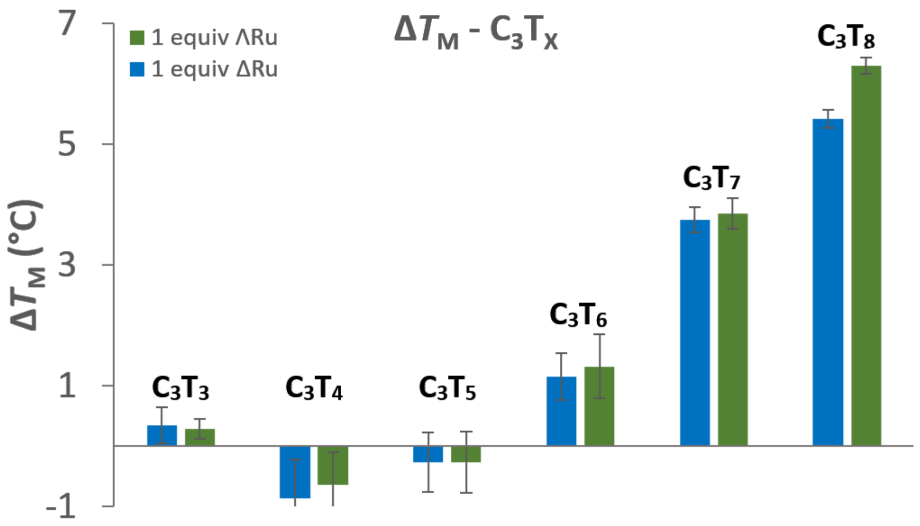

Figure S3.7. Comparison of the ΔT_M induced by Λ-Ru (green) and Δ-Ru (blue) for C_3T_X. Oligonucleotide concentration was 1 μM ss and ruthenium concentration was 1 μM. Buffer was 50 mM sodium cacodylate at pH 5, and data was obtained at 260 nm with a temperature interval of 1 °C.

4. Luminescence Data

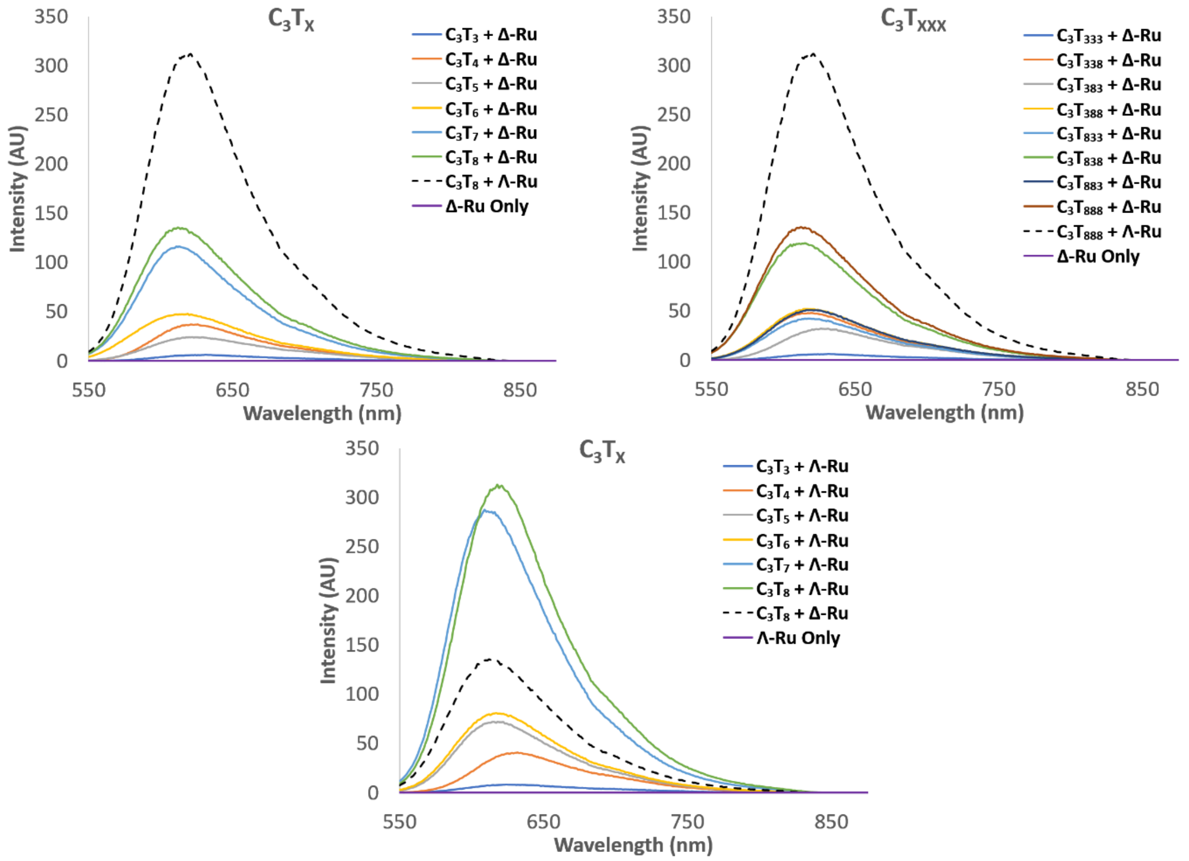

Figure S4.1. Top: Luminescence spectra of Δ-Ru (20 µM) bound with C_3T_X (left) or C_3T_{XXX} (right) at 20µM ss. The spectrum of Λ-Ru bound with C_3T_{888} is also shown for comparison (broken line). Bottom: Luminescence spectra of Λ-Ru (20 µM) bound with C_3T_X at 20µM ss. The spectrum of Δ-Ru bound with C_3T_{888} is also shown for comparison (broken line). Buffer consisted of 50 mM sodium cacodylate at pH 5. The excitation wavelength was 440 nm.

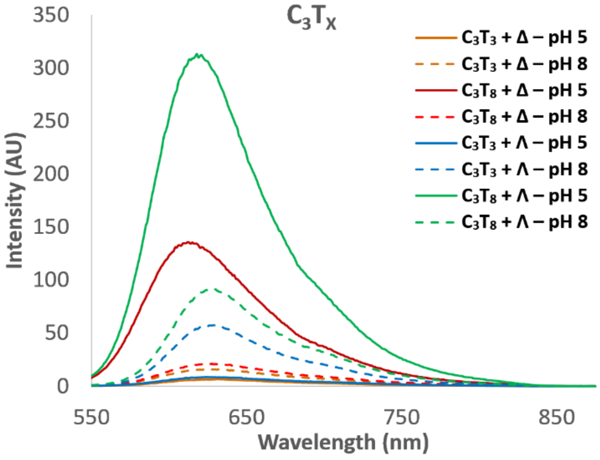

Figure S4.2. Luminescence spectra of Λ-Ru (blue and green) and Δ-Ru (orange and red), bound with C_3T_3 and C_3T_8, each at 20 µM concentration. Buffer consisted of 50 mM sodium cacodylate at either pH 5 (solid line) or pH 8 (broken line). Excitation wavelength was 440 nm.

Table S4.1. Luminescence data summary for the solutions of Λ-Ru and Δ-Ru (20 μM) bound with C_3T_X and C_3T_{XXX} (20 μM ss), conducted in 50 mM sodium cacodylate at pH 5 (unless mentioned otherwise). The "integral" value was determined through an approximation of the area under the luminescence curve. Values for the Ru complexes only are only approximations due to such a low signal without the presence of DNA.

**Label**	**Sequence 5'→3'**	**Λ-Ru**			**Δ-Ru**		
		**λ_{max} (nm)**	**Peak Intensity (AU)**	**Integral (AU^2)**	**λ_{max} (nm)**	**Peak Intensity (AU)**	**Integral (AU^2)**
Ru Complex Only	N/A	601	0.48	45	589	0.45	39
C_3T_3	$(CCCTTT)_3CCC$	624	8.4	956	631	6.9	738
C_3T_4	$(CCCTTTT)_3CCC$	632	40.9	4068	626	37.4	3654
C_3T_5	$(CCCTTTTT)_3CCC$	618	72.4	7128	622	24.4	2686
C_3T_6	$(CCCTTTTTT)_3CCC$	617	81.4	8107	619	48.3	5166
C_3T_7	$(CCCTTTTTTT)_3CCC$	609	287.4	27081	613	116.7	11311
C_3T_8	$(CCCTTTTTTTT)_3CCC$	618	313.2	29767	612	135.5	13467
C_3T_{338}	CCCTTTCCCTTTCCCTTTTTTTTCCC	617	114.3	11100	620	48.0	4934
C_3T_{383}	CCCTTTCCCTTTTTTTTCCCTTTCCC	629	319.8	27160	627	48.0	4934
C_3T_{388}	CCCTTTCCCTTTTTTTTCCCTTTTTTTTCCC	626	282.2	25453	617	52.5	5344
C_3T_{833}	CCCTTTTTTTTCCCTTTCCCTTTCCC	620	94.1	9211	618	42.7	4408
C_3T_{838}	CCCTTTTTTTTCCCTTTCCCTTTTTTTTCCC	616	271.3	25137	615	119.2	12054
C_3T_{883}	CCCTTTTTTTTCCCTTTTTTTTCCCTTTCCC	626	225.3	20456	616	51.3	5228
C_3T_3 (pH 8)	$(CCCTTT)_3CCC$	629	57.5	5316	632	15.8	1755
C_3T_8 (pH 8)	$(CCCTTTTTTTT)_3CCC$	627	91.3	8434	627	21.1	2266

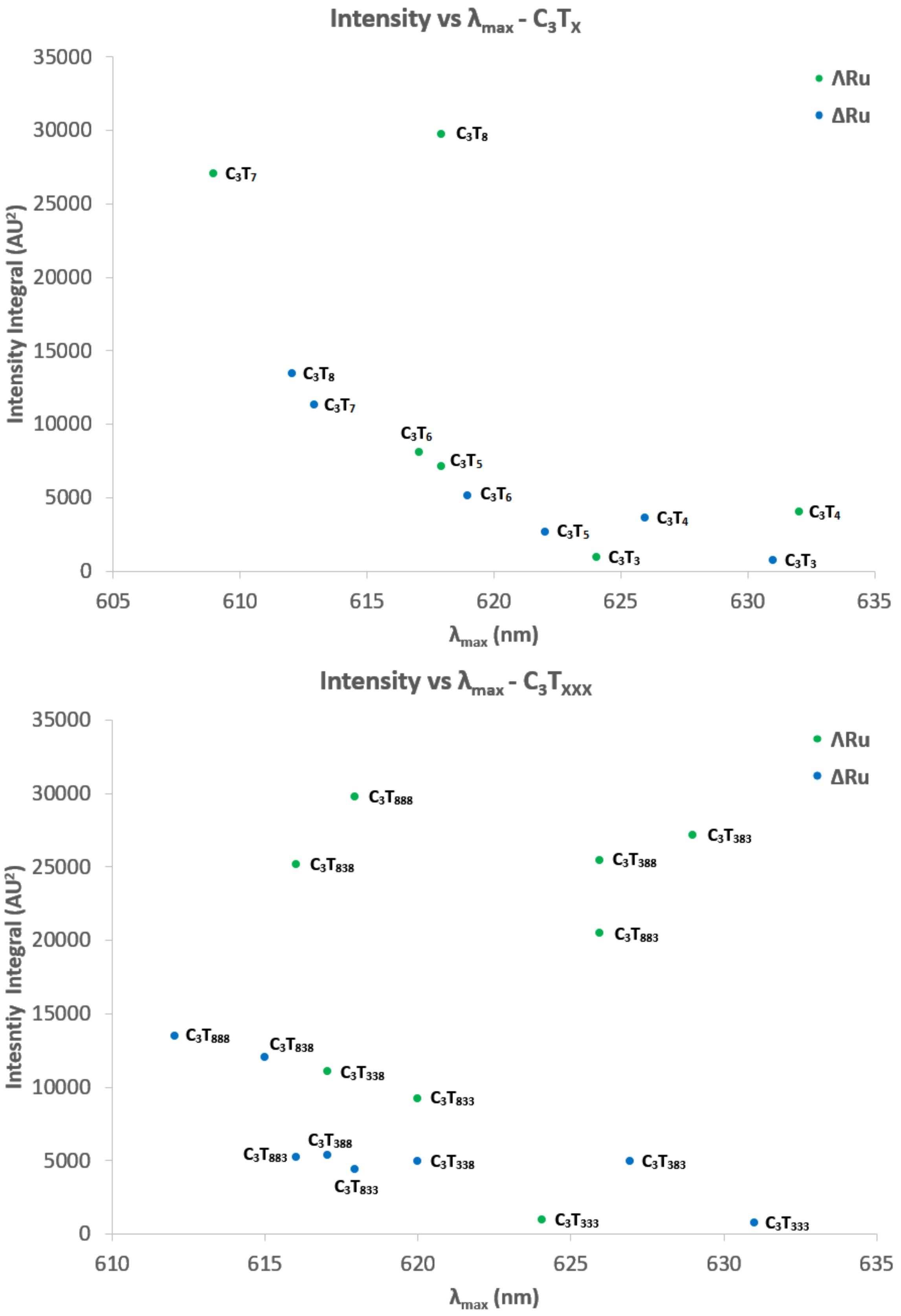

Figure S4.3. Comparison of luminescence intensity integral and λ_{max} of Λ-Ru (green, 20 µM) and Δ-Ru (blue, 20 µM) when bound to C_3T_X (top, 20 µM) and C_3T_{XXX} (bottom, 20 µM). Buffer consisted of 50 mM sodium cacodylate at pH 5. The excitation wavelength was 440 nm.

5. Melting Temperature vs Luminescence Data

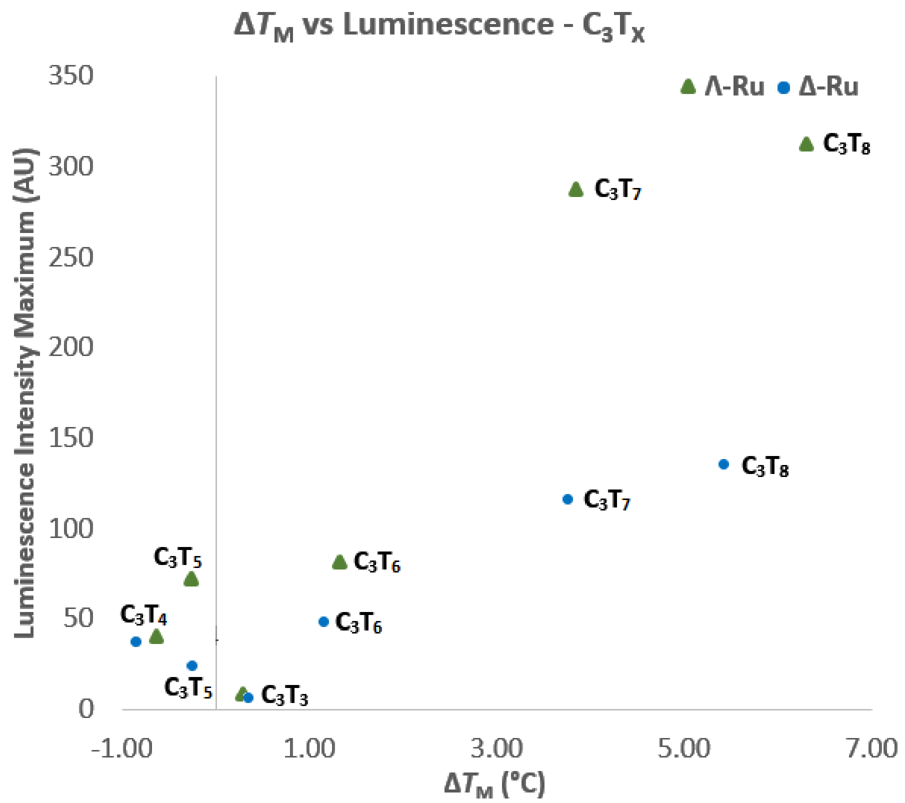

Figure S5.1. Comparison of the luminescence intensity maxima and ΔT_M of Λ-Ru (green) and Δ-Ru (blue) when bound with C_3T_X. Conditions are as elsewhere stated in this document.

6. References

Mcquaid, K., Abell, H., Gurung, S.P., Allan, D.R., Winter, G., Sorensen, T., et al. (2019). Structural Studies Reveal Enantiospecific Recognition of a DNA G-Quadruplex by a Ruthenium Polypyridyl Complex. *Angew. Chem., Int. Ed.* 58, 9881-9885. doi: 10.1002/anie.201814502
